# Does grafting of left anterior descending artery with right internal mammary artery have an impact on late outcomes in the context of multiple arterial grafting?

**DOI:** 10.1186/1749-8090-8-S1-O202

**Published:** 2013-09-11

**Authors:** S Raja, U Benedetto, M Husain, R Soliman, F De Robertis, M Amrani

**Affiliations:** 1 Department of Cardiac Surgery, Harefield Hospital, London, UK

## Background

Universally, anastomosis of the left internal mammary artery (LIMA) to the left anterior descending artery (LAD) is accepted as the gold standard in coronary artery bypass grafting. The use of the right internal mammary artery (RIMA) for grafting the LAD has recently emerged as an alternative to avoid composite configuration in multiple arterial grafting using bilateral internal mammary arteries. Whether this recently emerged alternative strategy of RIMA to LAD is as effective as the well-established gold standard LIMA to LAD remains unknown. We investigated the impact of using RIMA for grafting of LAD on late outcomes in the context of multiple arterial grafting.

## Methods

Among 1255 patients undergoing first time isolated CABG with multiple arterial grafting at a single institution, RIMA to LAD was used in 496 cases and LIMA to LAD in 759 cases. A propensity score adjusted analysis was carried out to investigate the impact of RIMA to LAD on late mortality and need for repeat revascularization.

## Results

Mean follow-up time was 4.6±2.9 years. In the propensity score adjusted analysis, the need for repeat revascularization was comparable for RIMA to LAD versus LIMA to LAD (HR 0.75; 95%CI 0.41-1.35; P=0.34) as well as the risk for late death (adjusted HR 0.77; 95%CI0.51-1.15; P=0.20). RIMA to LAD was associated with a non significant trend towards a lower rate for the composite outcome of death or need for repeat revascularization (adjusted HR 0.70; 95%CI 0.49-0.999; P=.05).

## Conclusions

RIMA to LAD is a valid alternative to LIMA to LAD while performing multiple arterial grafting. Avoiding composite graft configuration by using in situ double IMA might result in better late outcomes. Further studies with longer follow-up are needed to confirm these findings.

